# Up-regulation of sex-determining region Y-box 9 (SOX9) in growth hormone-secreting pituitary adenomas

**DOI:** 10.1186/s12902-021-00720-x

**Published:** 2021-03-18

**Authors:** Farzad Izak Shirian, Mohammad Ghorbani, Mohammad E. Khamseh, Mehrnaz Imani, Mahshid Panahi, Alimohammad Alimohammadi, Mitra Nourbakhsh, Vahid Salimi, Masoumeh Tavakoli-Yaraki

**Affiliations:** 1 Department of Biochemistry, School of Medicine, Iran University of Medical Sciences, Tehran, Iran; 2 Endocrine Research Center, Institute of Endocrinology and Metabolism, Iran University of Medical Sciences (IUMS), Tehran, Iran; 3 Firozgar Hospital, Pathology Department, Iran University of Medical Sciences, Tehran, Iran; 4 Iranian Legal Medicine Organizations, Tehran, Iran; 5 Department of Virology, School of Public Health, Tehran University of Medical Sciences, Tehran, Iran

**Keywords:** Growth hormone -pituitary adenoma, SOX9, Invasive tumors, Macroadenoma, Microadenoma

## Abstract

**Background:**

Pituitary adenomas are benign brain tumors that cause considerable morbidity and neurological symptoms. SOX9 as a regulatory transcriptional mediator affects normal and tumor cell growth with an undefined role in pituitary adenomas pathogenesis. Thus, in the present study, the expression pattern of SOX9 in GH-secreting pituitary tumors and normal pituitary tissues is investigated.

**Methods:**

The SOX9 gene expression level was evaluated in 60 pituitary tissues including different types of GH-secreting adenomas and normal pituitary tissues through Real-Time PCR. The protein level of SOX9 was assessed using immunohistochemistry. The correlations of SOX9 gene and protein expression level with the patient’s clinical and pathological features were considered.

**Results:**

The SOX9 over-expression was detected in GH-secreting adenomas tumor tissues compared to normal pituitary tissues which were accompanied by overexpression of SOX9 protein in tumor tissues. The over-expression of SOX9 had a significant impact on GH-secreting adenomas tumor incidence with the odds ratio of 8.4 and the diagnostic value of SOX9 was considerable. The higher level of SOX9 expression was associated with invasive and macro tumors in GH-secreting pituitary adenoma patients. The positive correlation of SOX9 gene and protein level was observed and the tumor size and tumor invasive features were valuable in predicting SOX9 expression level in GH-producing pituitary tumors.

**Conclusion:**

The study provided the first shreds of evidence regarding the expression pattern of SOX9 in the GH- secreting pituitary adenomas at both gene and protein levels which may emphasize the possible involvement of SOX9 as a mediator in pituitary adenoma tumor formation also open up new intrinsic molecular mechanism regarding pituitary adenoma pathogenesis.

## Background

Among the intracranial brain tumors, pituitary adenomas are common, complex, and multifactorial tumors that impose a great burden of morbidity on patients worldwide [1]. Growth hormone (GH)-secreting adenoma belongs to the hormone-secreting pituitary adenomas with insidious and debilitating clinical presentation ranging from calcium-phosphate alteration, the elevation of bone turn over, coarse facial features, and growth hormone over production [2, 3]. Most recently, the GH-secreting adenoma prevalence was reported as 2–13/100000 in the population with the approximate equal rate of incidence in males and females that mostly diagnosed over the age of 50. The clinical manifestations of patients besides excess growth hormone and Insulin-like growth factor-1 (IGF-I) levels are attributed to the diagnosis of the disease [4]. However, the diagnostic delay and limited early diagnosis in those patients lacking the main clinical features of GH-secreting adenoma likewise acral enlargement or those who suffer from overlapped conditions, make the disease consequences even more deleterious [5, 6]. The aberrant GH-producing cell proliferation in the anterior pituitary gland caused tumor formation which is mostly categorized as macroadenoma and triggers acromegaly clinical demonstrations [4, 7]. Accordingly, evidence revealed that pituitary tumor formation can be influenced by cancer stem cell (CSC) population likewise the presence of progenitor mesenchymal cells (PMCs) as CSC derivatives in GH-secreting adenoma and hormone in-active pituitary tumors [8–10]. Notably, the higher percentages of endothelial progenitor cells (EPCs) in the GH-secreting adenoma patients and their correlation with IGF-1was meaningful [11]. The sex-determining region Y-box 9 (SOX9) is a multi-functional transcription factor that is involved in tissue organogenesis, cell development, chondrogenesis, cell differentiation through its ability to direct cells to the specific lineage [12]. Regarding the notion of cancer stem cells (CSCs) which ease the cell re-newel and differentiation, recently SOX9 proposed as a CSC marker due to its ability to maintain cells in the undifferentiated status [13]. Notably, the correlation of SOX9 with cellular signaling pathway likewise NOTCH and Wnt, spotted this transcription factor as an appealing effector tumor cell fate [14]. The overexpression of SOX9 was reported in some types of malignancies such as metastatic melanoma [15] non-small cell lung cancer [16], prostate cancer [17], and esophageal squamous cell carcinoma [12]. It was shown that SOX9 beside the estrogen receptor mediates transcription of the collagen gene following stimulation of 17β-estradiol and induces chondrogenesis [18]. The feedback circuits between SOX9, Foxa2, and Tcf2 coordinate a network leading to the pancreatic progenitor cell programming and liver organogenesis [19]. In light of the SOX9 regulatory role in progenitor cell differentiation, it was revealed that following activation of SOX9 through the Notch pathway, it can activate the expression of downstream mediators mainly neurogenin 3, and induce pancreatic endocrine differentiation [14]. The presence of stem cells in the pituitary gland contributes to both pituitary organ regeneration also flexible differentiation to specific hormone-secreting cells in response to the body requirement [8]. Recent evidences are in favor of connecting pituitary stem cells to the process of pituitary tumor cell formation, growth, and development [20]. Given the potential role of SOX9 in tissue regeneration as well as its relevance to the intracellular pathways regulating tumor cell growth, this study is aimed to evaluate the status of SOX9 in tumor tissues of GH- secreting pituitary adenomas at both gene and protein level to provide pieces of evidence regarding the relevance of this effector with pituitary tumors. We applied both tumors and healthy tissues of the pituitary to provide a clear comparison.

## Methods

### Patients and sample collection

A total number of 60 pituitary samples including 40 GH-producing pituitary adenomas and 20 cadaveric healthy pituitary tissues were enrolled in the current study with local ethical approval and informed consent. The project was ethically approved by the ethics committee of the vice president of research at Iran University of Medical Sciences. Patients with GH-producing pituitary adenomas were enrolled from January 2017 to May 2019 and patients with a known history of any malignancy were excluded from the study. Also, to avoid the possible effect of pituitary adenomas medical treatments on SOX9 expression, only patients who have received no treatments before surgery were selected and enrolled in this study. All patients had been diagnosed by imaging and post-surgery pathology also patients received no radiotherapy before surgery when we took the samples. In this study, the patients subjected to the endoscopic transnasal transsphenoidal surgery (ETSS) at the neurosurgery department of our institute were participated and following surgical resection. Tumor samples were obtained and divided into two sections as follows, one part was transferred to the pathology department for further histological evaluations and the rest were immediately transferred to the lab in a cool situation for further evaluations. The healthy pituitary tissues were collected from the Legal Medicine Organization (LMO), Tehran, Iran at the first hour of their death. The total number of 20 human healthy pituitary autopsies was collected from cases with no pathological pituitary problems from January 2017 to May 2019. The anterior epithelial lobe of pituitary tissues was selected without histological evidence of pathological features. Normal pituitary autopsies were matched to the enrolled patients as a matter of age and gender. The mean healthy donor’s age range was 46.52 ± 0.3 and an equal number of male and female donors participated in the study. Also, 85% of healthy donor’s Body mass index (BMI) was ≤25 while 15% was over 25. The tumor and normal pituitary sample collection and processing was followed as our previous studies [21, 22]. The clinic- pathological features of patients with pituitary adenoma are summarized in Table 1. As is mentioned, amongst the patients with GH- pituitary adenomas, 55% of the patients were female and 45% were male, also the invasive feature of the tumor was detected in 35% of patients while the non-invasive tumor was diagnosed in 65% of patients It should be noticed that invasive pituitary tumors were classified based on Knosp classification system in which tumor extension to the adjacent sphenoid sinus and cavernous sinus is defined as invasive tumor and also, confirmed by the neurosurgeon during the endoscopic surgery. Based on our patient’s characters, the mean tumor size of the patients was 14.25 ± 1.43 that the type of tumor in 70% of our patients was macroadenoma but in 30% of patients the tumor type was microadenoma. Notably, in our study, the tumor body greater than 10 mm in size was considered as macroadenomas and the tumor body less than 10 mm in size was defined as microadenomas. Additionally, the mean patient’s age was 47.88 ± 4.2 which 42.5% of our patients were below 40 years of age, while 57.5% were over 40 years of age, respectively. Also, 32.5% of healthy donor’s body mass index (BMI) was ≤25 while 67.5% of patients had BMI over 25. The statistical analysis regarding participants ‘age, gender and, BMI revealed that the difference between the BMI of patients and healthy controls was statistically significant. The patient’s biochemical features and hormone levels were assessed and summarized in Table 2.

**Table 1:** The clinicopathological features of patients with GH-producing pituitary adenoma

Parameter	Groups	Patients (N, %)	Healthy subjects (N, %)	*P*-Value
Age at diagnosis (Years)	≤40	17 (42.5%)	10 (50%)	0.5820
	40≤	23 (57.5%)	10 (50%)	
Gender	Female	22 (55%)	10 (50%)	0.7874
	Male	18 (45%)	10 (50%)	
Body mass index (BMI)	≤25	13 (32.5%)	17 (85%)	0.0003
	25≤	27 (67.5%)	3 (15%)	
Tumor invasiveness ^a^	Invasive	14 (35%)		
	Non- Invasive	26 (65%)		
Tumor size^b^	Micro-Adenoma	10 (30%)		
	Macro-Adenoma	30 (70%)		

^a^ The definition of invasive tumor is presented in **“**Patients and sample collection” section

^b^ The definition of macro/micro adenoma is presented in “Patients and sample collection” section

**Table 2:** The biochemical features of patients with GH-producing pituitary adenoma

Parameters	Normal range	Standard deviation ± Mean
Blood Sugar (mg/dl)	Normal: 100–145	156.1 ± 13.2
Alkaline phosphatase (U/L)	Normal: Male 80–306 Female 64–306	228.5 ± 23.7
Calcium (Serum) (mg/dL)	Normal: 8.6–10.3	141 ± 13.8
Sodium (Serum) (mmol/L)	Normal: 136–145	142.4 ± 1.13
Inorganic Phosphorus (mg/dL)	Normal: 2.7–4.5	5.8 ± 0.83
Potassium (mmol/L)	Normal: 3.5–5.5	4.04 ± 0.11
Prolactin (mIU/L)	Normal: Male: 87–392 Female 132–498	691 ± 30 909 ± 61
Growth Hormone (ng/ml)	Women:0.126–9.88 Men:0.03–2.47	14.71 ± 5.01 6.64 ± 2.3
ACTH (pg/ml)	7.2–63.3	40.49 ± 7.18
Cortisol (μg/dl)	6.2–19.4	21.20 ± 5.5
IGF-1 (ng/ml)	53–234	675.9 ± 68.62
Testosterone (ng/ml)	Men:2.5–10 women:0.2–0.95	1.17 ± 0.37 0.3 ± 0.1
Age		47.88 ± 4.2
Tumor size		14.25 ± 1.43

### RNA extraction, cDNA synthesis, real-time PCR

To evaluate the expression level of SOX9, the tissue RNA was extracted and subjected to assessment via Real-Time PCR. Accordingly, the RNA content of tumor and healthy pituitary tissues were extracted using Trizol (Invitrogen, Grand Island, USA) based on the manufacturer’s instructions. Briefly, tissues were lysed and homogenized with 700 μL of Trizol lysis reagent and following an adequate incubation period, chloroform was added to each tissue homogenate for subsequent phase separation. The upper, aqueous phase was collected and transferred to the new tube and mixed with isopropanol. After an appropriate incubation time and centrifugation, the supernatant was discarded and the RNA pellet washed with 75% ethanol. The RNA pellet was air-dried and dissolved in an appropriate volume of RNase-free water. The extracted RNA of each tissue was evaluated as a point of quality and quantity with a Nanodrop spectrophotometer (Nanodrop Technologies). The total amount of 1 μg of RNA was applied for cDNA Synthesis via PrimeScript First Strand cDNA Synthesis Kit (Takara, Japan) according to the manufacturer’s instructions. The gene expression was assessed via Real-Time PCR using the SYBR Premix Ex Taq II (Takara, Japan) via Applied Biosystems Step One Plus, Real-time system (Applied Biosystems, USA). The expression running time and program was as follow: 1 cycle at 95 °C for 5 min following 40 cycles at 95 °C for 5 S, 55 °C for 20 S, and 60 °C for 35 S. Beta-actin was used as a housekeeping gene for SOX9 gene expression normalization due to its constitutive expression pattern in the pituitary gland that was applied in previous reports [23–25]. The formula RQ = 2^-ΔCT^ was used to analyze the relative gene expression levels after normalization with the endogenous controls and the SOX9 and beta-actin melt curves were considered as primer specificity. The sequence and characters of primers were as: SOX9 forward primer: *5′– GCTTCTCGCTCTCGTTCAGA-3′*, SOX9 reverse primer: *5′-CAACGGCTCCAGCAAGAACA − 3′* (Tm = 58), beta-actin forward primer: *5′-GAT CTC CTT CTG CAT CCT GT-3′*, beta-actin reverse primer: *5′-TGG GCA TCC ACG AAA CTA C- 3′* (Tm = 57).

### Immunohistochemistry

The 4 μm tissue section of 40 paraffin-embedded pituitary adenoma samples were applied for immunohistochemistry analysis using an anti- SOX9 monoclonal antibody (Cell Signaling, the Netherlands) as the primary antibody. The slides were subjected to de-paraffinization and rehydration, subsequently followed by getting immersed in methanol containing 0.3% hydrogen peroxide for 20 min to inhibit peroxidase activity endogenously. The antigen retrieval was performed using citrate buffer incubation for 10 min. The dilution of 1:400 of the primary antibody of SOX9 was applied following proper incubation; the secondary antibody (Cell Signaling, the Netherlands) was added afterward. To visualize the antigen/antibody reaction the 3, 3′-diaminobenzidine (DAB, Dako) was used. The slide scoring was assessed by two independent pathologists who were blinded to the data and discrepancies between them were resolved by consensus. Based on the antibody included protocol the colon cancer tissue was taken as a positive control. For evaluation of SOX9 immunoreactivity, the results were divided into four graded according to the percentages of positive cells and staining intensity as follows: 0; less than 5% of neoplastic cells were stained indicating absence of immunoreactivity, 1+; 5–25% of neoplastic cells were stained indicating weak intensity staining, 2+; 26–50% of neoplastic cells were stained indicating moderate intensity staining, and 3+; more than 50% of neoplastic cells were stained indicating strong intensity of staining. Depending on the cellular density of the tumor, the number of tumor cells ranged from 200 to 300 cells per field that assessed in in five different hot spot areas [26, 27].

### Statistical analysis

For gene expression analysis the comparative CT (2^-ΔCt^) method was applied wherein ΔCt corresponds to the Ct of the SOX9 subtracted from the Ct of the endogenous gene (Beta-Actin). The Kolmogorov-Smirnov analysis was applied to determine the normal distribution of data in patients and normal groups. Due to the normal distribution, a t-test was used to analyze the difference between the expression of SOX9 between pituitary tumor and normal tissues. The receiver operating characteristic (ROC) curves and calculation of area under the curve (AUC) were used to determine the diagnostic value of SOX9 expression in tumor and normal tissue [28, 29]. To categorize the SOX9 expression level, a cut-off value was determined using receiver operating characteristic (ROC) analysis. A cut-off value was indicated as the value of SOX9 that maximized the sensitivity and specificity of the discrimination between pituitary tumor and normal tissue based on the Youden Index [28]. Tumor tissues with SOX9 expression levels below the cut-off were classified as low expression tumors, and those with SOX9 expression levels above the cut-off were classified as high expression. The chi-square test was performed to calculate the correlation of SOX9 expression levels (i.e., low or high expression) with patients’ clinicopathological characteristics (age, gender, Body mass index (BMI), tumor size, and invasive status). The association and possible impact of SOX9 expression as a risk factor for GH-secreting pituitary adenoma was calculated using the odds ratio [30]. The regression analysis was applied to determine the value of the variables in predicting SOX9 expression level in tumors. The correlation between SOX9 gene expressions with SOX9 protein immunohistochemistry intensity staining also Ki67 labeling index was analyzed using Pearson coefficient test in patients with GH-producing pituitary adenoma. The enumeration data are presented as percentages. Between-group comparison of enumeration data, the chi-square test was used. The Graph Pad Prism version 6 (Graph Pad Software, San Diego California) and Statistical Package for Social Science (SPSS v.20) were used for the calculation of all statistics. *P* values < 0.05 (two-sided) were considered statistically significant.

## Results

### The expression level of SOX9 was increased in GH- secreting pituitary adenoma

**Fig. 1:**
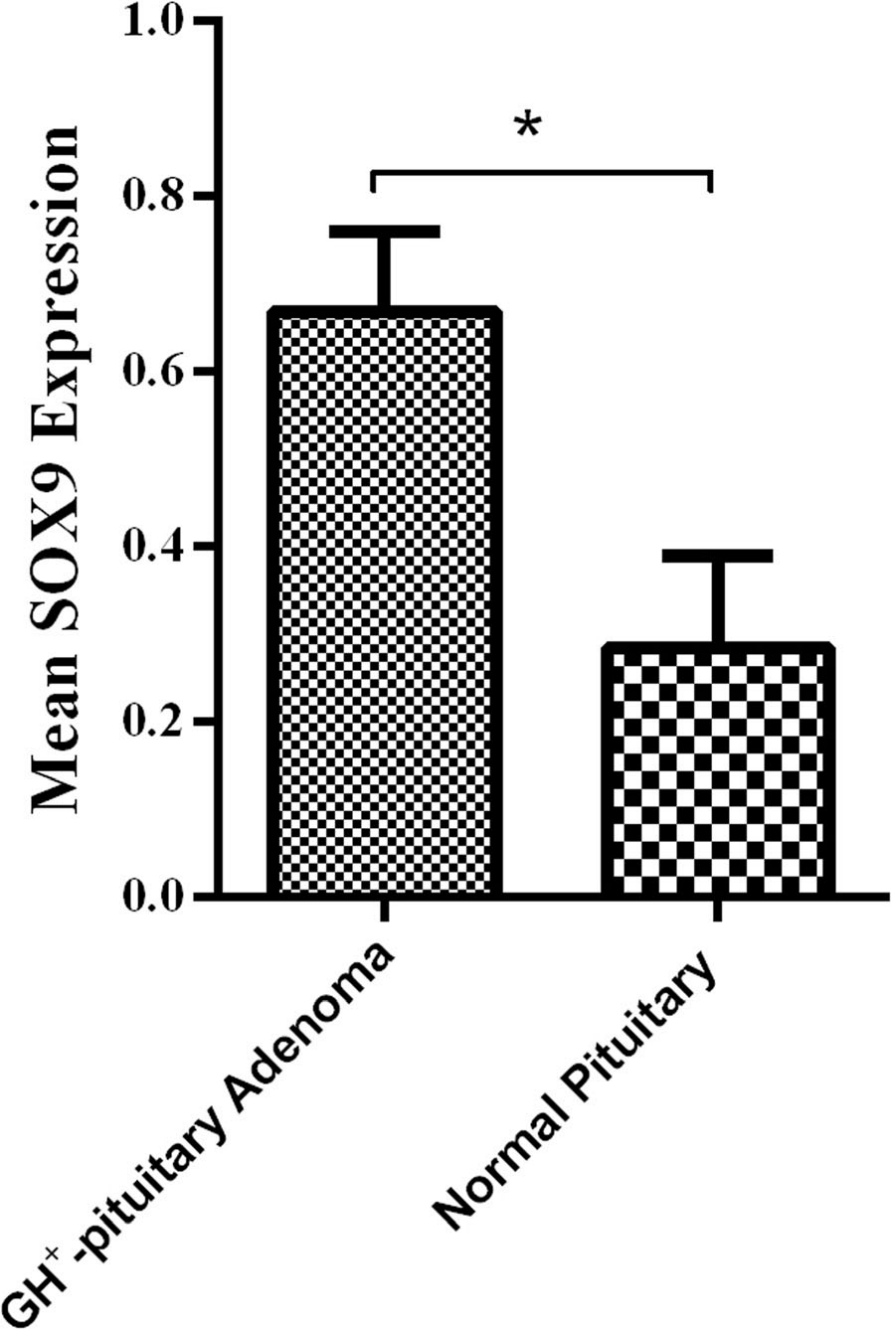
The SOX9 expression level in GH-secreting pituitary adenomas. The expression level of SOX9 was assessed in GH-secreting pituitary tumors and normal pituitary tissues. The SOX9 gene expression level was elevated in GH-secreting pituitary adenoma. The Statistical differences between groups are shown as an asterisk (* = *P* < 0.05)

**Fig. 2:**
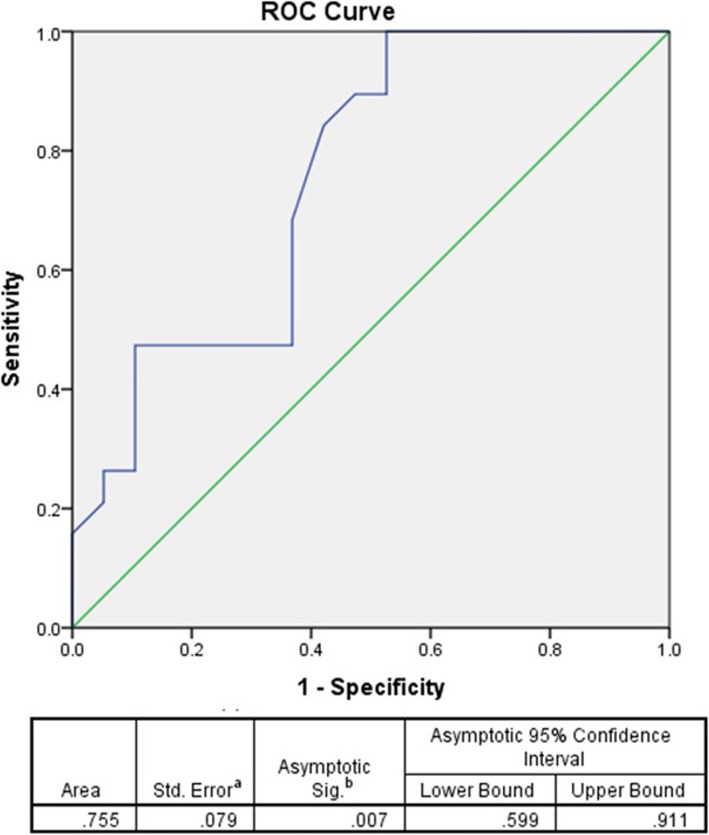
The SOX9 gene expression ROC Curve. The SOX9 gene expression ROC Curve, area under the curve, 95% confidence interval, *P*-value is shown

According to our data, the SOX9 expression level was enhanced significantly in tumor tissues comparing to normal pituitary tissues (*P* = 0.019) (Fig. 1). The mean and standard error mean (SEM) of SOX9 mRNA level was 0.66 ± 0.020 and 0.28 ± 0.105 in patients and control group, respectively. The SOX9 expression was detected around 2.3 times more in tumor tissues comparing to healthy pituitary tissues. Moreover, to determine the strength of association between SOX9 tumor expression level and GH- secreting pituitary adenoma, the odds ratio was calculated that it was revealed that SOX9 tumor expression level significantly affect adenoma incidence by the odds ratio of 8.4 (*P* = 0.015) (Table 3). To categorize the SOX9 expression, the receiver operating characteristic (ROC) curve was applied and a cut-off value was determined. The value with the maximized sensitivity and specificity was selected as a cut-off that was able to discriminate between tumor and normal pituitary adenoma. The tumors with SOX9 expression level below the cut-off value were defined as low expression tumors and the tumors with the SOX9 expression level over the cut-off value were categorized as high expression levels. Based on our data, the ROC curve of SOX9 expression in GH-producing pituitary tumor and normal pituitary tissues revealed the AUC level of 0.755 (95% CI, 0.599–0.911, *P* = 0.007) with the best cut off value of 0.54 which has 84.5 and 60% sensitivity and specificity, respectively based on the Youden Index (Fig. 2). As is shown in Table 4, 58.82% of patients below 40 years of age revealed a low expression level of SOX9, while 41.17% of them expressed a higher level of SOX9. Also, 43.47 and 56.52% of patients over 40 years of age showed a low and high levels of SOX9 expression, respectively. Based on data presented here, the correlation of age with SOX9 tumor expression was not significant in GH- secreting pituitary adenoma patients. Besides, 54.54% of female patients expressed a low SOX9 level while 45.45% of them expressed a high level of SOX9 in tumor tissues. However, 44.44 and 55.55% of male patients with GH- secreting pituitary adenoma tumors presented low and high SOX9 expression levels, respectively. In accordance, the patient’s gender showed no significant correlation with the SOX9 level of expression. Among the invasive GH- secreting tumors 35.71 and 64.28% of tumors expressed low and high SOX9 levels, respectively. However, 69.23% and 30,76% of non-invasive tumors presented low and high SOX9 tumor expression, respectively. Based on the analysis, the correlation of tumor invasive features with SOX9 tumor expression was significant. Also, tumor size revealed a positive correlation with SOX9 expression level since 70% of patients with microadenoma showed low SOX9 expression while 30% of patients revealed high SOX9 tumor expression. However, 26.66 and 73.33% of patients with macroadenoma showed low and high SOX9 tumor expression, respectively. Notably, the Ki67 is a non-histone nuclear protein that accounts as a marker of cell proliferation that is expressed throughout the active phase of the cell cycle [31]. In the present study, the immunoreactivity of the Ki67 was detected and the positivity was reported as a percentage. Based on the results, none of the enrolled patients illustrated the Ki67 index higher than 3% while the mean Ki67 proliferation index was 0.4828 ± 0.02953 in non-invasive tumors and 1.468 ± 0.1908 in invasive tumors. The difference in mean Ki67 proliferation index between invasive and non-invasive GH- secreting pituitary adenoma was statistically significant (*P* = 0.001). The correlation between SOX9 expression and the Ki67 proliferation index in invasive and non-invasive tumors was analyzed using the Pearson coefficient test which shows no significant correlation (*P* = 0.55, *P* = 0.28, respectively). The regression analysis of the common variables in GH- secreting pituitary adenomas demonstrated that the tumor size and tumor invasive feature were significantly valuable in predicting SOX9 tumor expression while the predictive value of patients age, gender, Growth hormone, and IGF-1 level was not statistically significant (Table 5).

**Table 3:** Associations between SOX9 expression and GH-producing pituitary adenoma

	B	S.E.	Wald	df	*P* value	Exp(B)	95% C.I.for EXP(B)	
							Lower	Upper
SOX9	2.134	0.874	5.966	1	0.015	8.451	1.525	46.84

**Table 4:** Associations between SOX9 expression and clinicopathological features of the patients

Parameter	Groups	*SOX9* expression				*P*-Value
		High expression		Low expression		
		%	N	%	N	
Age (Years)	≤40	41.17%	7	58.82%	10	0.52
	40≤	56.52%	13	43.47%	10	
Gender	Female	45.45%	10	54.54%	12	0.75
	Male	55.55%	10	44.44%	8	
Tumor invasiveness	Invasive	64.28%	9	35.71%	5	0.052
	Non- Invasive	30.76%	8	69.23%	18	
Tumor size	Micro-Adenoma	30%	3	70%	7	0.023
	Macro-adenoma	73.33%	22	26.66%	8

**Table 5:** The regression model of SOX9 tumor expression in patients with GH-producing pituitary adenoma

Variable (predictors)	Standardized Coefficients (Beta)	t	*P* value
Patient age	0.200	1.16	0.25
Patient gender	−0.322	−1.19	0.064
Tumor size	0.400	3.47	0.001
Tumor invasion	0.350	2.73	0.01
Growth hormone level	0.033	0.21	0.835
IGF-1 hormone level	−0.296	−1.85	0.072

### The SOX9 protein level increased in tumor tissues of GH-producing pituitary adenomas

**Fig. 3:**
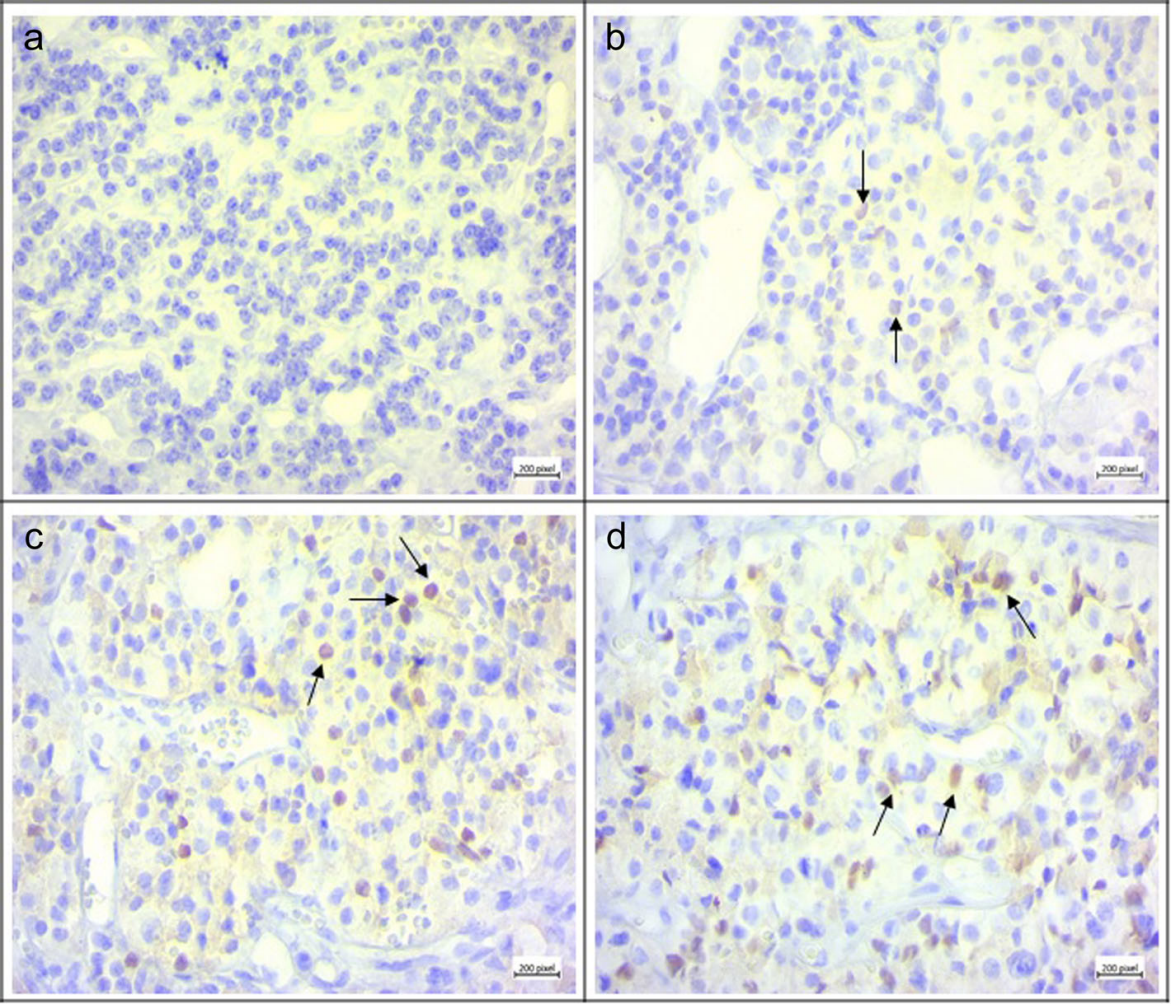
The SOX9 immunohistochemistry. SOX9 differential protein expression was evaluated by immunohistochemistry in GH-secreting tumor tissues. **a** Normal pituitary tissue. The score 0; < 5% of neoplastic cells staining, absence of immunoreactivity, × 200., **b** GH-secreting tumor tissues. 1+; 5–25% of neoplastic cells staining, weak intensity of staining × 200. **c** GH-secreting tumor tissues. 2+; 26–50% of neoplastic cells staining, moderate intensity staining, × 200. **d** GH-secreting tumor tissues.2+; 26–50% of neoplastic cells staining, moderate intensity staining, × 200

**Fig. 4:**
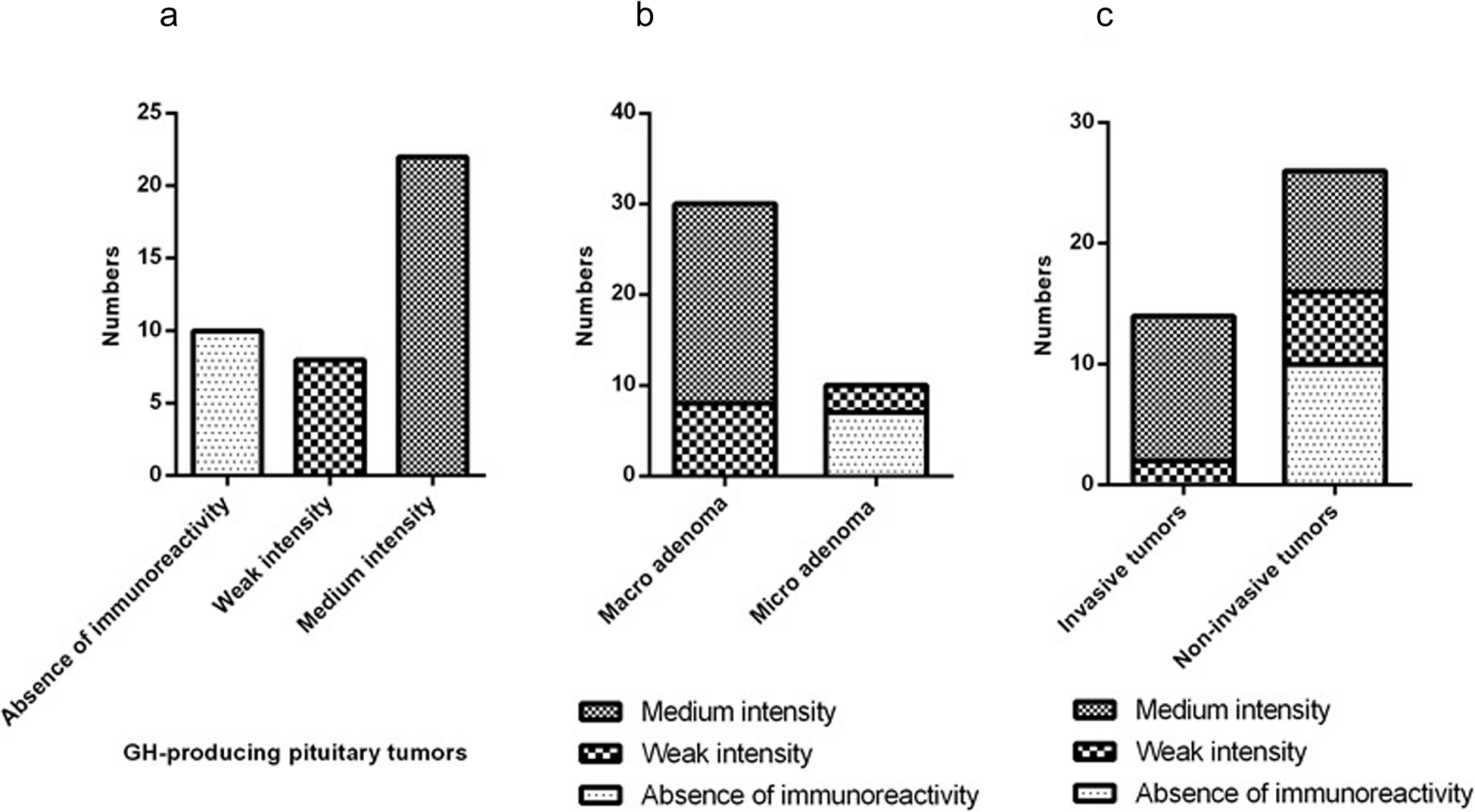
Comparison of SOX9 expression in different types of GH-secreting pituitary tumors using immunohistochemistry. Bar graphs show the staining intensity of SOX9 in different types of GH-secreting pituitary tumors. Different bar pattern represents the intensity of staining that are described under the graph. X-axis represents the intensity of staining (**a**) and type of GH-secreting pituitary tumors (**b**, **c**). Y-axis represents the number of patients positive for a particular type of intensity. **a**, **b**, and **c** showing expression patterns of SOX9 in GH-secreting pituitary tumors, macroadenoma vs microadenoma, invasive vs. non-invasive tumors, respectively

Based on our results, SOX9 protein was undetectable in normal pituitary tissues. In addition, 25% of GH-producing pituitary tumors showed no expression of SOX9 while 20 and 55% of tumors revealed a weak and moderate SOX9 protein expression (Score 1+, Score 2+), respectively (Table 6). Based on the scoring method, none of the tumor sections revealed strong and intensive expression of SOX9 in tumors. The strong expression revealed the pattern that more than 50% of cells in the tissue section were positively stained with the SOX9 antibody. It was revealed that all of the macroadenoma tumors expressed SOX9 protein that amongst, 26.66% of the macroadenoma tumors expressed weak (Score1+) and 73.33% of macroadenoma tumors expressed moderate (Score 2+) SOX9 protein. While negative SOX9 protein expression was detected in 70% of microadenoma tumors and weak SOX9 expression was detected in 30% of microadenoma tumors (Score 1+). The difference in the SOX9 protein expression level between macro and micro-GH-secreting pituitary adenoma was statistically significant (*P* < 0.0001). Based on our data, 14.28% of invasive GH-producing pituitary tumors showed weak expression of SOX9 protein expression (Score 1+), while 85.71% of invasive tumors revealed a moderate level of SOX9 protein (Score 2+). However, 38.46% of non-invasive GH-producing pituitary tumors showed no expression of SOX9 protein while 23.07 and 38.46% of non-invasive tumors showed weak and moderate expression of SOX9 protein (Score 1+, Score 2+, respectively). The difference in the SOX9 protein expression level between GH-secreting invasive and non-invasive pituitary adenoma was statistically significant (*P* = 0.009). Moreover, the representative images of SOX9 immunohistochemistry staining in GH-producing pituitary adenoma tumor tissues are illustrated in Fig. 3 and the comparison of SOX9 protein expression in different types of GH-secreting pituitary tumors is shown in Fig. 4. Besides, to evaluate the possible relevance of SOX9 gene expression with SOX9 protein expression level, a correlation between SOX9 gene expression in different tumor subtypes and its protein expression was evaluated and the results are demonstrated in Table 7. Based on data, the SOX9 gene expression in GH-secreting pituitary tumors was significantly correlated with the SOX9 protein level (*P* = 0.007), also, in the subgroup of macroadenoma, the SOX9 gene expression was significantly correlated with its protein expression level (*P* = 0.022) while no correlation was observed regarding the SOX9 gene and protein level in microadenoma group. Regarding invasive pituitary tumors, it was revealed that the SOX9 gene expression was significantly correlated with its protein expression level (*P* = 0.01), while the correlation between SOX9 gene and protein expression was not significant in non-invasive tumors (Table 7).

**Table 6:** The immunohistochemistry results of SOX9 protein evaluation in patients with GH-producing pituitary adenoma

Groups	Expression pattern ^a^							*P* value
	Total number	Score 0		Score 1+		Score 2+		
		N %		N %		N %		
GH-producing pituitary tumors	40	10	25%	8	20%	22	55%	
Macro adenoma	30	0	0	8	26.66%	22	73.33%	< 0.0001
Micro adenoma	10	7	70%	3	30%	0	0	
Invasive tumors	14	0	0	2	14.28%	12	85.71%	0.009
Non-invasive tumors	26	10	38.46%	6	23.07%	10	38.46%	

^a^ The Expression pattern was determined based on the staining intensity of SOX9 antibody that is described in material and methods

**Table 7:** The correlation of SOX9 gene expression with SOX9 protein level in patients with GH-producing pituitary adenoma

Variable	Pearson Coefficient test	SOX9 protein expression
**SOX9 gene expression in Tumor**	Correlation	0.4248
	*P*-value	0.007
**SOX9 gene expression in Macro adenoma**	Correlation	0.4160
	*P*-value	0.022
**SOX9 gene expression in Micro adenoma**	Correlation	0.0537
	*P*-value	0.8828
**SOX9 gene expression in Invasive tumors**	Correlation	0.6576
	*P*-value	0.0106
**SOX9 gene expression in Non-invasive tumors**	Correlation	0.2959
	*P*-value	0.1421

## Discussion

Pituitary adenomas represent around 15% of brain tumors with the heterogeneous types of neoplasms which the GH-secreting adenomas account as one of the most prevalent forms of functional pituitary adenomas [2, 32, 33]. The dynamic of pituitary gland cells is tightly related to the body’s hormonal requirements which steer the vital process likewise growth, reproduction, development, and immune responses [34–37]. In support of this, characterizing the profile of pituitary adenoma biomarkers might be helpful both as a point of prognostic and predictive approach also as a matter of mechanism underlying pituitary tumor genesis although their functional role in pituitary pathogenesis is debatable. More recently, the relevance of SOX9 to cell development, growth, and differentiation has received more attention due to its role to maintain cells in the undifferentiated status [13, 38, 39]. Moreover, it was reported that the reduction of SOX9 was correlated with a decrease in the ability of prostate cancer cells to migrate and proliferation [17]. Additionally, the overexpression of SOX9 in non-small cell lung cancer was correlated with tumor size and tumor severity [16]. Since the status of SOX9 in pituitary adenoma tumors was not determined, the expression pattern of this mediator in GH-secreting pituitary adenoma was investigated in the current study. Based on our data, the higher expression of SOX9 was detected in tumor tissues comparing to normal pituitary tissues. To the best of our knowledge, no similar study was implemented in pituitary tumors although its overexpression was reported in other neoplasms such as melanoma [15] non-small cell lung cancer [16], prostate cancer [17],and esophageal squamous cell carcinoma [12]. Due to our data, SOX9 was expressed more in macroadenoma comparing to tumors with small size. Also, the relevance of SOX9 with pituitary tumor size was confirmed by the assessment of SOX9 protein level in our patients’ tumor sections, and it was revealed that all of the macroadenoma pituitary tumors expressed SOX9 protein. Accordingly, the invasive GH-producing pituitary tumors expressed a higher level of SOX9 comparing to non-invasive tumors also the higher protein level of SOX9 was observed in invasive tumors. Based on data, the SOX9 protein level was significantly correlated with the SOX9 gene expression level in the subgroup of macroadenoma, microadenoma, and non-invasive GH-secreting pituitary adenoma. As mentioned earlier, the Ki67 index accounts as a marker of cell proliferation that is expressed throughout the active phase of the cell cycle [31]. Based on the European Society of Endocrinology guidelines, the Ki67 labeling index higher than 3% can be considered as a positive prognostic marker in pituitary adenoma. However, the real value of Ki67 and the use of this criterion as an indicator of pituitary tumor aggressiveness is controversial and the views in this regard are vague due to different methodologies and definitions of tumor progression [40]. The Ki67 proliferation index shows a significant difference between invasive and non-invasive GH- secreting pituitary adenomas while it was not correlated with SOX9 expression. Even though pituitary adenomas account as benign and slow-growing tumors, while the expression profile of SOX9 in these tumors obeys the same pattern as SOX9 demonstrated in more malignant solid tumors such as lung and prostate [16, 17]. In our study, no specific differences were observed in the level of SOX9 expression in male and female participant’s tumor tissues although the correlation of SOX9 with gender in pituitary adenomas can be clarified by further studies. In the present study, no difference was observed in the level of SOX9 in different age groups, therefore the association of SOX9 with age in patients with pituitary tumors cannot be concluded in the study. Based on recent evidences SOX9 mediates activation of neurogenin 3 through the Notch pathway which leads to pancreatic endocrine differentiation [14]. Besides, exogenous SOX9 induces chondrogenesis, differentiation, and proliferation of adipose-derived mesenchyme stem cells as well as nervous system, lung, and heart organogenesis [41]. Besides its role in inducing cell differentiation to the specific lineage, SOX9 can maintain cells in the undifferentiated status as it is observed in suppressing epidermal differentiation in hair follicle stem cells [42]. Also, it was shown that SOX9 ^+^ cells derived from human hepatocellular carcinoma (HCC) were highly proliferative and able to self-renewal which makes them an appropriate CSC marker in HCC [43]. In line with the evidences, the over-expression of SOX9 in GH- secreting pituitary tumors unravel the possible contribution of this regulatory transcription factor in pituitary tumor formation also differentiation to GH secreting cells however this claim should be clarified by more mechanistic studies. As it was observed from our results, SOX9 over-expressed in GH-secreting tumor tissues and the differences in its expression level were significant between tumor subtypes such as macro adenoma tumors and invasive GH-producing pituitary tumors. However, based on the nature of pituitary adenoma tumors which are mainly benign and the limited number of participants with invasive tumors in our study, extending and investigating the role of SOX9 in GH-producing pituitary adenoma tumorigenesis in more diverse patients are recommended.

## Conclusions

Our study provides first data regarding the status of SOX9 as an appealing marker in patients with GH-secreting pituitary adenoma. Due to our data, the overexpression of the SOX9 gene level was observed in pituitary tumor tissues in comparison to normal tissues which was associated with an elevated level of SOX9 protein in tumor tissues. In GH-secreting pituitary tumor tissues as prevalent pituitary tumors, the increased level of SOX9 was observed in tumors with bigger in size and invasive features which is in line with the impressive role of SOX9 in cell proliferation and invasion. The presence and overproduction of SOX9 as a pivotal stem cell marker in GH-secreting pituitary adenoma may highlight the effective impact of stem cells in the pituitary gland which might facilitate organ regeneration also cell differentiation to hormone-secreting cells. Also, it might provide shreds of evidence regarding the possibility of SOX9 involvement in pituitary adenoma tumor formation that can delineate the GH-secreting pituitary adenoma underlying molecular mechanism.

## Data Availability

All data generated or analyzed during this study and supporting our findings are included and can be found in the manuscript. The raw data can be provided by the corresponding author on reasonable request.

## Abbreviations

SOX9: Sex-determining region Y-box 9
IGF-I: Insulin-like growth factor-1
GH: Growth hormone
CSC: Cancer stem cell
PMCs: Progenitor mesenchymal cells
EPCs: Endothelial progenitor cells
SSTR: Somatostatin receptors
ETSS: Endoscopic transnasal transsphenoidal surgery
LMO: Legal Medicine Organization
DAB: 3, 3′-diaminobenzidine
HCC: Human hepatocellular carcinoma
ROC: Receiver operating characteristic
AUC: Area under the curve
